# 10 mm (PI-RADS v2.1) versus 15 mm (PI-RADS v1.0) tumor capsule contact length in predicting extracapsular extension in prostate cancer: Meta-analysis and systematic review

**DOI:** 10.1007/s00261-025-04998-6

**Published:** 2025-06-05

**Authors:** Dheeman Futela, Mahima Bhargava, Sharath Rama, Sishir Doddi, Yong Chen, Nikhil H Ramaiya, Sree Harsha Tirumani

**Affiliations:** 1 University Hospitals Cleveland Medical Center, Cleveland, USA; 2 Shorewood High School, Shoreline, WA, USA; 3 Beth Israel Deaconess Medical Center, Boston, USA; 4 University of Toledo, Toledo, USA

**Keywords:** Magnetic resonance imaging, Meta-analysis, Prostate cancer, Extraprostatic extension

## Abstract

**Objective:**

To determine the diagnostic performance of tumor capsule contact length (TCCL) and to determine its optimal cut-off on prostate MRI to predict extra-capsular extension (ECE) at histopathology of radical prostatectomy specimen in patients with prostate cancer.

**Materials and methods:**

An electronic search of the PUBMED and EMBASE databases was performed until December 31, 2024 to identify studies analyzing TCCL as a predictor of ECE on prostate MRI. Pooled sensitivity and specificity of all studies were plotted in a hierarchical summary receiver operating characteristic plot and the risk of ECE was evaluated using bivariate random-effects meta-analysis. Meta-regression analysis for various TCCL cut-offs was performed.

**Results:**

Twenty-three eligible studies were found. Reported sensitivity (between 47 − 94%) and specificity (between 46 − 88%) showed significant heterogeneity between studies, without evident threshold effect. Pooled sensitivity and specificity of TCCL for predicting ECE was 76.3% and 68.8% respectively with AUC of 0.787 for the summary ROC curve. Meta-regression analysis showed no significant difference in diagnostic performance according to various TCCL cutoffs, measurement methods, or ISUP grade distribution. Studies with 14 mm threshold had similar sensitivity (73%), but greater specificity (74% vs. 70%) and diagnostic odds ratio (8.3 vs. 6.5), when compared to 10 mm threshold.

**Conclusion:**

TCCL on MRI has reasonable pooled sensitivity and specificity to predict ECE. While sensitivity remained moderately high for all TCCL thresholds, specificity at 14 mm (cutoff close to 15 mm suggested by PI-RADS v1.0) threshold was greater than that at 10 mm (cutoff suggested by PIRADS v2.1). This cutoff can be helpful in developing objective likelihood scores for ECE prediction on MRI.

## Introduction

The management of prostate cancer depends on accurate staging of both local and distant spread [1]. When prostate cancer is confined to the prostate (≤ T2c) without extracapsular extension (ECE) (T3a), seminal vesicle invasion (T3b) or metastatic disease, curative treatment is possible [2]. While serum PSA, digital rectal examination and transrectal ultrasound guided biopsy allow assessment of aggressiveness of prostate cancer, local staging of prostate cancer needs more robust techniques for definitive treatment planning. Multi-parametric MRI is increasingly used in the management of prostate cancer both for detecting clinically significant prostate cancer and for local staging [3–6]. Several studies have investigated the diagnostic accuracy of MRI in local staging of prostate cancer [4, 7–9]. A large meta-analysis of 75 studies found that MRI has high specificity but poor and heterogeneous sensitivity for local staging of prostate cancer [8]. In this study, the sensitivity and specificity of MRI for ECE (T3a), seminal vesical invasion (T3b) and overall T3 disease was 57–61% and 88–96% respectively^8^. While high specificity for an imaging technique is preferable to avoid false positives and unnecessary over treatment, a high sensitivity in prostate MRI may be preferable to urologists to decrease number of positive surgical margins and preserve neurovascular bundles. Accordingly, there is a need to improve the diagnostic performance of MRI in accurate staging of prostate cancer.

**Fig. 1:**
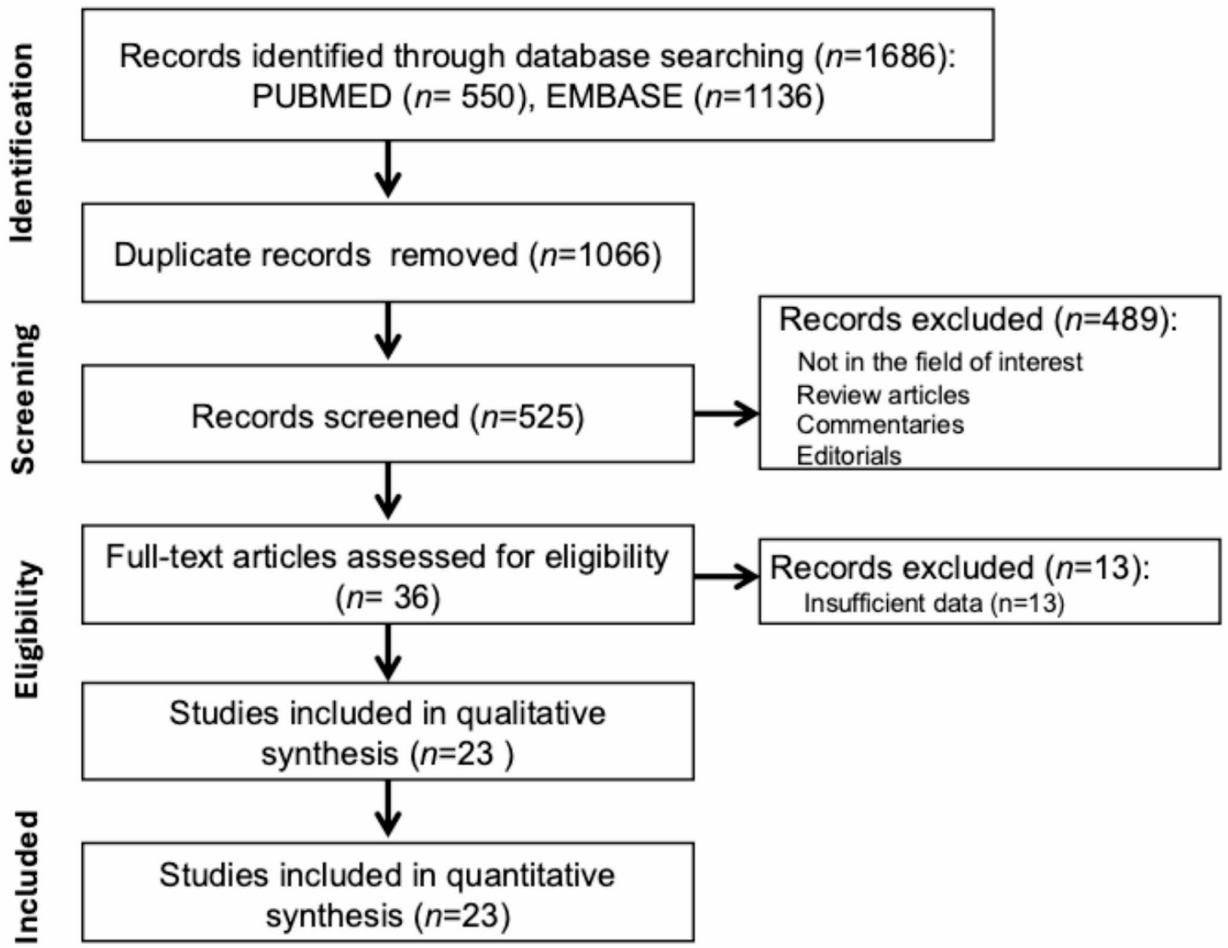
Preferred Reporting Items for Systematic Reviews and Meta-Analyses (PRISMA) flow diagram showing study selection process for the meta-analysis

The European Society for Urogenital Radiology proposed in 2011 criteria and scoring for ECE on MRI which included capsular contact (1 point), capsular irregularity (3 points), neurovascular bundle thickening or capsular bulge (4 points) and overt capsular spread (5 points) [10]. A score of ≥ 4 increases likelihood of ECE. Of these criteria, tumor capsule contact has garnered interest with several studies evaluating it as a potential indicator of ECE mainly due to the subjective nature of the rest of the criteria (Fig. 1). Tumor capsule contact length (TCCL) refers to length of contact between the dominant tumor focus and the prostatic capsule. A long TCCL can predict ECE when other standard criteria are not apparent [11, 12]. In the PI-RADS version 1.0, a broad capsule contact of greater than > 1.5 cm on T2 weighted imaging was assigned a score of 5 for peripheral zone lesions. In the PI-RADS version 2.0/2.1, a tumor capsule abutment of greater than 1 cm is mentioned as a staging criterion. However, several studies in the recent literature have proposed different cut-offs for TCCL ranging from as low as 6 mm to as high as 24 mm to predict ECE [11–35]. Given these various thresholds in published studies, it is necessary to evaluate the existing literature to determine the optimal TCCL threshold. A meta-analysis published in the year 2020 including 13 studies between 1994 and 2019 concluded that increasing TCCL increased the probability of ECE but did not propose an optimal threshold as there was no difference in specificity and sensitivity for 10 mm and 12 mm as cut offs [36]. Since the year 2020, several original articles have investigated the diagnostic performance of MRI in predicting ECE with some focusing on TCCL. Accordingly, the objective of this meta-analysis was to validate the overall diagnostic performance of TCCL in predicting ECE and to determine the optimal cut-off of TCCL on prostate MRI which can predict ECE at histopathology of radical prostatectomy specimen in patients with prostate cancer.

## Methods

The Preferred Reporting Items for Systematic Reviews and Meta-Analyses (PRISMA) guidelines were followed in designing this meta-analysis [37]. The research question formulated according to the PICOS criteria was: what is the diagnostic performance of TCCL and its optimal cut-off on prostate MRI to predict ECE at histopathology of radical prostatectomy specimen in patients with prostate cancer?

### Data sources, search strategy and selection criteria

We performed electronic search of two databases, PUBMED and EMBASE between January 1, 2000 and December 31, 2024, to identify studies analyzing TCCL as a predictor of ECE on prostate MRI. The following search query containing combinations of prostate cancer, was used for the search: ((“prostat* cancer”) OR (“prostat* carcinoma”) OR (“prostat* neoplasm”) OR (“prostat* tumor”)) AND ((“magnetic resonance imaging”) OR (“MR imaging”) OR (MRI) OR (MR)) AND ((“extraprostatic extension”) OR (“extracapsular extension”) OR (“EPE”) OR (“capsule contact”)). The search was limited to studies on human subjects and those published in English language. All the identified articles and their bibliographies were manually searched to find relevant articles meeting the inclusion criteria.

To be eligible, the studies had to meet the following inclusion criteria: (1) included patients with suspected or pathologically proven prostate cancer who were being staged for prostate cancer and were treatment naïve (2) included patients underwent radical prostatectomy (3) had histopathology of radical prostatectomy as reference standard (4) used at least T2-weighted imaging for staging (5) included patients with MRI visible contact between tumor and capsule and reported diagnostic performance of TCCL with a specified cutoff and (6) were original research articles. The studies were excluded if they were considered review articles, guidelines, consensus statements, editorials, conference abstracts. Studies which did not have radical prostatectomy specimen as histopathology conformation of prostate cancer, had insufficient data for reconstruction of 2 × 2 contingency tables for calculating sensitivity and specificity and studies that used TCCL as part of a grading system or used qualitative criteria (like capsule irregularity or bulging) for determining ECE were also excluded. The literature search, eligibility of the studies which fulfilled the inclusion and exclusion criteria was reviewed (xx (blinded for review)) by one of the study team members.

### Data extraction and quality assessment

The study, patient and index test characteristics were extracted from each of the included studies. The study characteristics included authors, publication year, study period, institution, single or multicenter study, prospective or retrospective study, consecutive versus non-consecutive enrollment, the reference standard used (pathology) and clinical outcome assessed. The patient characteristics which were obtained included number of patients, age of the patients (mean or median age in years), the inclusion criteria for each study, interval between prostate biopsy and MRI, interval between MRI and surgery, PSA level (mean or median), surgical pathology Gleason score, presence of extra-prostatic extension and seminal vesical invasion. For index test characteristics information about magnet strength and vendor/model, use of endorectal coil, administration of anti-peristaltic agent, sequences and imaging plane reviewed, slice thickness, number of readers, experience of readers, type of read (independent versus consensus), whether readers were blinded to the histopathology, sequence and the type of tool used for measuring the capsule contact length were recorded. Two study team members (yy and zz (blinded for review)) in consensus extracted data from the included studies. The quality of the included studies was assessed by another reader (xx blinded for review) using the quality assessment of diagnostic accuracy studies (QUADAS-2) questionnaire [38].

### Data synthesis and statistical analysis

The primary endpoint assessed in this meta-analysis was the diagnostic performance of TCCL and its optimal cutoff in predicting ECE on MRI in patients with prostate cancer. Descriptive analysis was performed to report mean or median for continuous variables. Heterogeneity across the included studies was determined using Cochranes’s chi-squared test (if *p* < 0.05, heterogeneity was explored). To evaluate the threshold effect, which is a positive correlation between sensitivity and the false-positive rate, visual assessment of coupled forest plot as well as a Spearman correlation were performed. A Spearman coefficient of > 0.6 indicates the presence of a threshold effect. In studies reporting results of multiple independent readers, the results of the most accurate reader were used. The pooled estimates of sensitivity and specificity of TCCL for predicting ECE in all studies was assessed using a bivariate random-effects meta-analysis [39]. The diagnostic odds ratio was defined as the odds of having ECE in patients with TCCL compared with the odds of having ECE in patients without tumor capsule contact. A hierarchical summary receiver operating characteristic (HSROC) curve was plotted with 95% confidence interval. Meta-regression analysis for various TCCL cut-offs was performed. Publication bias was studied using Deeks’ funnel plot asymmetry [40].

All statistical analysis was performed by two reviewers (xx and yy (blinded for review), with 2 years of experience in performing systematic reviews and meta-analyses) on the Revman 5.3 (The Nordic Cochrane Center, The Cochrane Collaboration, Cophenhagen) and “mada” library in R Studio v 2024.09.1.

## Results

The systematic literature search yielded 1686 articles. After removing 1066 duplicates, manual screening of the titles and abstracts of the remaining 525 studies identified 36 original research articles which evaluated TCCL as a predictor of EPE. The full text of these 36 articles was reviewed to exclude 13 studies due to insufficient data for reconstruction of 2  × 2 tables. The remaining 23 studies consisting of 3634 patient sample size in total were included in the meta-analysis. Figure 1 depicts the process involved in study selection.

## Study characteristics

The study, characteristics are summarized in Table 1. The studies were published between 2014 and 2024. Twenty studies were retrospective and three were prospective in study design [16, 20, 23]. The studies were performed at a single institute in 21 articles and multi-institutional in 2 articles [20, 31]. The patient enrollment was specified as consecutive in 9 studies, and unspecified in the other 14. The reference standard in all the studies was the histopathology of the radical prostatectomy specimen.

**Table 1:** Study characteristics

First Author	Institution	Country	Duration of patient recruitment	Year of publication	Single institution or multi-institutional	Type (prospective vs. retrospective)	Consecutive enrollment
Woo	Seoul National University Hospital	Seoul, Republic of Korea	Jan to Dec 2012	2016	Single	Retrospective	NA
Costa	University of Texas Southwestern Medical Center	Texas, USA	Nov 2015 to Jul 2016	2018	Single	Retrospective	Yes
Dominguez	Hospital Universitario Fundación Santa Fe de Bogotá	Bogotá, Colombia	May 2011 to Dec 2013	2018	Single	Retrospective	Yes
Baco	USC Institute of Urology, Keck School of Medicine	California, USA	May 2011 to Dec 2013	2014	Single	Retrospective	Yes
Kongnyuy	National Cancer Institute	Maryland, USA	May 2007 to Dec 2015	2017	Single	Prospective	NA
Krishna	Ottawa Hospital, University of Ottawa	Ontario, Canada	Nov 2012 to May 2015	2017	Single	Retrospective	Yes
Matsumoto	Keio University School of Medicine	Tokyo, Japan	2011 to 2016	2019	Single	Retrospective	NA
Matsuoka	Ochanomizu Surugadai Clinic	Tokyo, Japan	Aug 2007 to Mar 2015	2017	Single	Retrospective	NA
Mehralivand	University Medical Center, Mainz	Mainz, Germany	Jun 2007 to Mar 2017	2019	Multiple	Prospective	Yes
Rosenkrantz	New York University	New York, USA	NA	2015	Single	Retrospective	NA
Rud	Oslo University Hospital	Oslo, Norway	Dec 2009 to Jun 2012	2018	Single	Prospective	NA
Onay	Baskent University School of Medicine	Ankara, Turkey	2012 to 2017	2019	Single	Retrospective	NA
Miyamoto	Hiroshima University Hospital	Hiroshima, Japan	Apr 2014 to Jul 2019	2021	Single	Retrospective	NA
Ayaz	Medeniyet University, Siirt Training and Research Hospital	Istanbul, Turkey	May 2014 to Aug 2017	2023	Single	Retrospective	NA
Eurboonyanun	Massachusetts General Hospital, Boston	Massachusetts, USA	Apr 2016 to Oct 2016	2021	Single	Retrospective	NA
Valentin	University Dusseldorf	Dusseldorf, Germany	Jan 2016 to Dec 2017	2021	Single	Retrospective	NA
Asfuroglu	Ankara Etlik City Hospital	Ankara, Turkey	Jan 2019 to Feb 2022	2024	Single	Retrospective	NA
Caglic	Norfolk & Norwich University Hospital, Norwich	Norfolk, UK	Sep 2014 to Jan 2017	2019	Single	Retrospective	NA
Bakir	Koc University Hospital, VKF American Hospital, and Istanbul Medical Faculty Hospital	Istanbul, Turkey	2012 to 2018	2019	Multiple	Retrospective	NA
Park	Asan Medical Center, University of Ulsan College of Medicine	Seoul, Republic of Korea	Jul 2016 to Mar 2017	2020	Single	Retrospective	Yes
Gatti	University of Turin - Presidio Ospedaliero Molinette	Turin, Italy	Jan 2015 to Dec 2020	2022	Single	Retrospective	Yes
Ito	Jichi Medical University Saitama Medical Center	Saitama, Japan	Jun 2014 to Jul 2019	2022	Single	Retrospective	Yes
Xu	Peking Union Medical College Hospital	Beijing, China	Jan 2017 and Jan 2021	2023	Single	Retrospective	Yes

Patient characteristics of included studies are summarized in Table 2. The number of patients in the studies ranged between 75 and 553 and the mean/median age between 61 and 69 years. Gleason scores or ISUP grade of the surgical pathology were reported in all but one study [13]. Prevalence of seminal vesicle invasion was reported in 8 studies [11, 14, 19, 20, 24, 25, 28, 32].

**Table 2:** Patient characteristics

First Author	Number of patients	Age (years)	PSA level (ng/mL)	Seminal Vesicle Invasion (number)	ISUP Grade (number)				
					I	II	III	IV	V
Woo	185	66.7 (45–79)	102 (0.5–123)	15	60	75	37	6	7
Costa	80	64 (46–78)	8 (1.8–46.3)	NA					
Dominguez	79	61.1 (39–78)	7 (0.02-31)	4	2	53		14	10
Baco	111	64 (45–75)	8.9 (2.5–44)	3	23	50	24	11	3
Kongnyuy	379	61 (47–75)	8.8 (0.2–53.5)	NA	301			78	
Krishna	149	62.3	6.33 (0.011–18.65)	NA	6	62	56	8	17
Matsumoto	233	65.8 (43–78)	Anterior tumor: 9.5, Posterior tumor, 8.6	NA	106		85	42	
Matsuoka	210	67 (50–81)	7.0 (2.9–30.0)	15	15	158		37	
Mehralivand	553	60 (52–68)	6.28 (5.02)	37	53	267	37	136	60
Rosenkrantz	90	64	9.0	NA	14	39	23	6	8
Rud	183	65 [IQR 60–68]	7.9 [IQR 5.8–11.5]	NA	57	73	21	25	7
Onay	105	62 (40–77)	7.95 (2.1–46)	NA	5	61	24	5	10
Miyamoto	91	69 [IQR 43–78]	7.3 [IQR 3.6–46.4]	6	25		66		
Ayaz	110	65.25 (48–79)	NA	NA	17	40	18	19	16
Eurboonyanun	95	62.4 (50–77)	6.34 (0.9–2.4)	NA	25	32	11	15	11
Valentin	136	67 [IQR 62–72]	9.3 [IQR 7–14]	38					
Asfuroglu	84	62.3 (45–79)	12.75	NA	33	27	10	3	11
Caglic	75	64.5 [IQR 52.7–67]	8.5 [IQR 5.7–10.4]	NA	6	40	12	8	9
Bakir	86	62.5	7.52 (2.1–40)	NA	3	48	19	5	11
Park	301	65 (35–82)	7.55 (0.32–42.9)	32	93	101	53	39	15
Gatti	100	66 [IQR 60–71]	7 [IQR 4.8–9.6]	NA	1	39	37	15	9
Ito	93	67.9 (51–78)	12.7 (2.7-121.6)	NA	2	41	16	6	28
Xu	141	65 [IQR 62–69]	9 [IQR 6.3-14.12]	NA	18	48	41	16	18

The MR specifications and index test characteristics are summarized in Table 3. The magnet strength was 3T in 16 studies, 1.5T in 6 studies and both in one study. Endorectal coil was used in 3 studies. All studies included at least T2-weighted and DWI/ADC sequences as part of their study design. T2-weighted images were used for measuring TCCL in all the studies. In addition, TCCL was measured on DCE sequence in 4 studies. A curvilinear tool was used to measure TCCL in 17 studies, linear tool in 3 studies and unspecified in 3. The number of readers varied from 1 to 5 with reader experience of 1–22 years.

**Table 3:** MRI and index test characteristics

First author	MRI			TCCL measurement				Readers		
	Vendor	Model	Magnet Strength	Sequence	Tool	Cutoff (mm)	Threshold method	Number of readers	Reader experience	Independent or consensus (if multiple readers)
Woo	Seimens, Philips	Magnetom Verio Trio; Ingenia	3T	T2, ADC, DCE	curvilinear	14	ROC	1	22 years	NA
Costa	Philips	Ingenia or Achieva with endorectal and phased-array surface coil	3T	T2W	linear	10	PIRADS v2	5	3 experienced (> 50 prostate MRIs), 2 inexperienced	Independent
Dominguez	Seimens	NA	1.5T	NA	NA	12	NA	2	14 years, 8 years	NA
Baco	Seimens	Avanto	1.5T	T2W	curvilinear	20	ROC	1	> 800 prostate MRIs	Independent
Kongnyuy	Phlips	Achieva, Philips Healthcare	3T	T2W	curvilinear	12.5	ROC	2	8 years, 16 years	NA
Krishna	Seimens, GE	TRO TIM and Discovery 750 W	3T	T2W	curvilinear	11	ROC	2	11 years, 15 years	Independent
Matsumoto	NA	NA	1.5T, 3T	T2W	curvilinear	10	NA	2	NA	NA
Matsuoka	Philips	Achieva, Philips Healthcare	1.5T	T2W	curvilinear	10	ROC	2	10 years, 5 years	Independent
Mehralivand	Philips	Achieva, Philips Healthcare	3T	NA	curvilinear	15	NA	2 (one per study)	15 years, 9 years	Independent
Rosenkrantz	Seimens	Magnetom Trio, Skyra or Biograph	3T	T2, ADC	linear	6	ROC	2	1 year, 4 years	Independent
Rud	Seimens	Avanto	1.5T	T2W	NA	14	ROC	1	1 year	Independent
Onay	Seimens	Magnetom Skyra	3T	T2, ADC, DCE	curvilinear	14	ROC	2	12 years, 5 years	Independent
Miyamoto	Philips	Ingenia	3T	T2W	curvilinear	14.1	ROC	1	NA	NA
Ayaz	GE	Optima 450 W	1.5T	T2W	curvilinear	9.5	ROC	2	4 years, 3 years	Independent
Eurboonyanun	GE	Discovery MR-750	3T	T2W, DCE	linear	15	ROC	2	5 years, 3 years	Consensus
Valentin	Seimens	Magnetom Trio TIM, Skyra	3T	T2W	curvilinear	11	ROC	3	10 years, 5 years, 2 years	Consensus
Asfuroglu	Seimens	Magnetom Verio	3T	T2W, DCE	curvilinear	15	NA	2	12 years, 6 years	Independent
Caglic	GE	MR750	3T	T2W	NA	13.5	ROC	1	8 years	NA
Bakir	Seimens	Magnetom Skyra	3T	T2W	curvilinear	15.2	ROC	2	6 years, 3 years	Independent
Park	Philips and Seimens	Ingenia and Sykra	3T	T2W	curvilinear	10	ROC	2	15 years, 3 years	Independent
Gatti	Philips	Achieva and Ingenia	1.5T	T2W	curvilinear	10	PIRADS v2	2	> 2000, 500 prostate MRIs	Consensus
Ito	Canon Medical Systems	Vantage Titan	3T	T2W	curvilinear	6.9	ROC	2	8 years, 9 years	Independent
Xu	GE Healthcare/Philips	GE750/Ingenia Elition	3T	T2W	curvilinear	20	ROC	1	> 1000 prostate MRIs	Independent

## Data quality

**Fig. 2:**
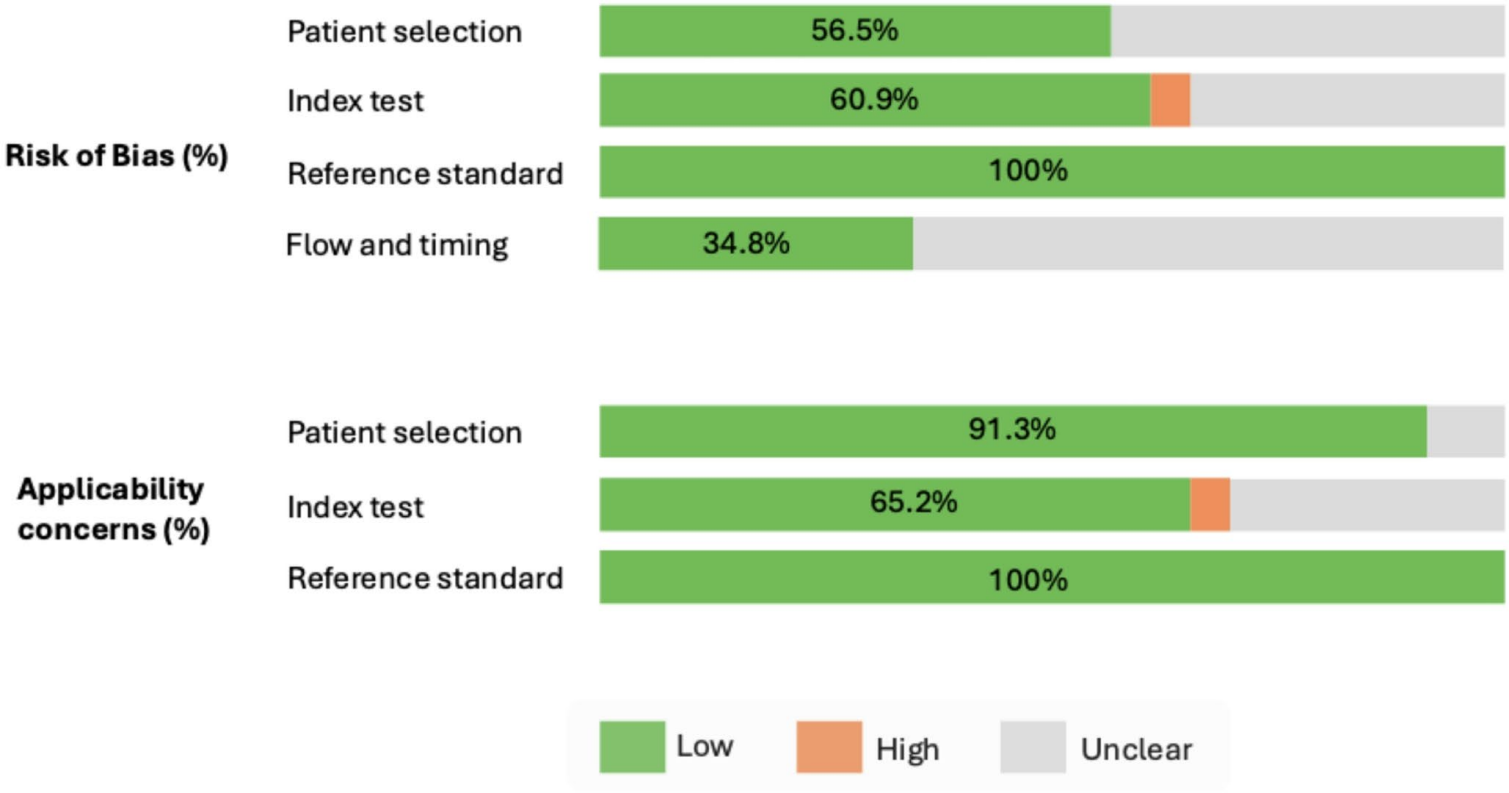
Summary of quality assessment of diagnostic accuracy studies (QUADAS-2) questionnaire results

Assessment of study quality using QADAS-2 questionnaire showed moderate quality of the studies. (Fig. 2) In the patient selection domain, the risk of bias was unclear because of lack of reporting on patient enrollment in ten studies [12, 16, 18, 19, 22–24, 26, 29, 31]. Applicability concern for patient selection was satisfactory for 21 studies but unclear in two studies [25, 28] due to non-reporting of inclusion or exclusion criteria. In the index test domain, the risk of bias and applicability was high in one study due to lack of reporting of slice thickness, reader experience or blinding of the reader as well as presence of a single reader for the study [11]. Risk of bias and applicability concerns for index test was unclear due to lack of reporting of blinding of readers in three studies [16, 23, 28], lack of reporting of reader experience in two studies [18, 25] and presence of single readers in three studies [20, 24, 35]. There was no risk of bias or applicability concern in reference standard domain as all the studies used histopathology of radical prostatectomy as reference standard. In the flow and timing domain, risk of bias was unclear due to lack of reporting of neither time interval between biopsy and MRI nor time interval between MRI and surgery in 15 studies [11, 14, 16–20, 25–27, 29, 32–35].

### Diagnostic performance

**Fig. 3:**
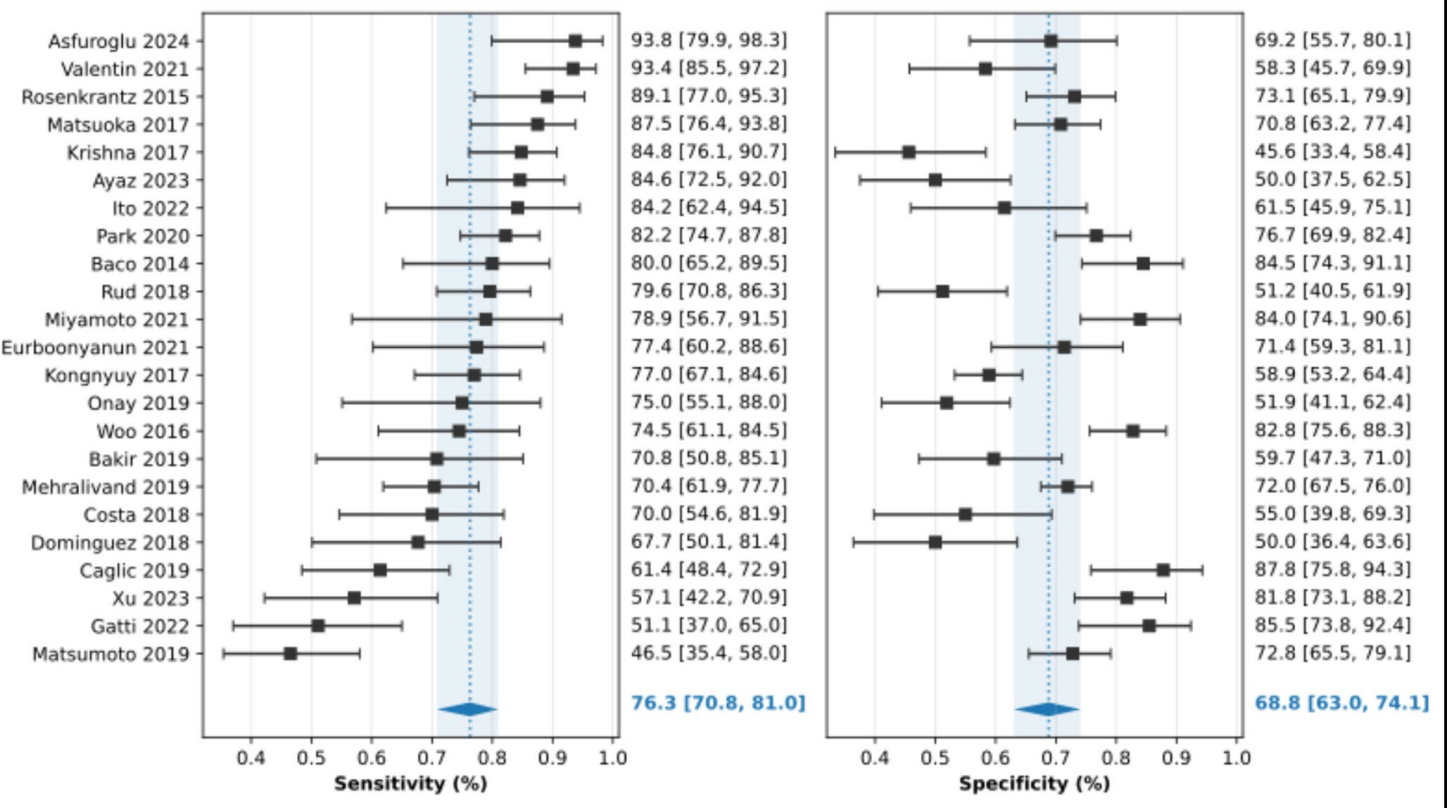
Coupled Forest plot of sensitivity and specificity. Horizontal error-bars indicate 95% confidence intervals

The TCCL cutoff used varied between 6 mm and 20 mm. A cutoff of 10 mm or less was used in eight studies, 11–15 mm in nine studies and ≥ 15 mm in 6 studies. The reported sensitivity of TCCL for predicting ECE for the 23 studies varied between 46.5% and 93.8%. The reported specificity varied between 45.6% and 87.8%. (Fig. 3) Significant heterogeneity was observed for both sensitivity and specificity, according to the coupled forest plots and chi-squared test for heterogeneity (df = 19, *P* < 0.001 for both). No threshold effect was evident by visual assessment of the coupled forest plot and the correlation between sensitivity and false positive rate was weak (Spearman correlation coefficient 0.336 [95% CI -0.09–0.66]). Area under the ROC curve (AUC) using quantitative TCCL was reported in 15 studies and varied between 0.67 and 0.89.

**Fig. 4:**
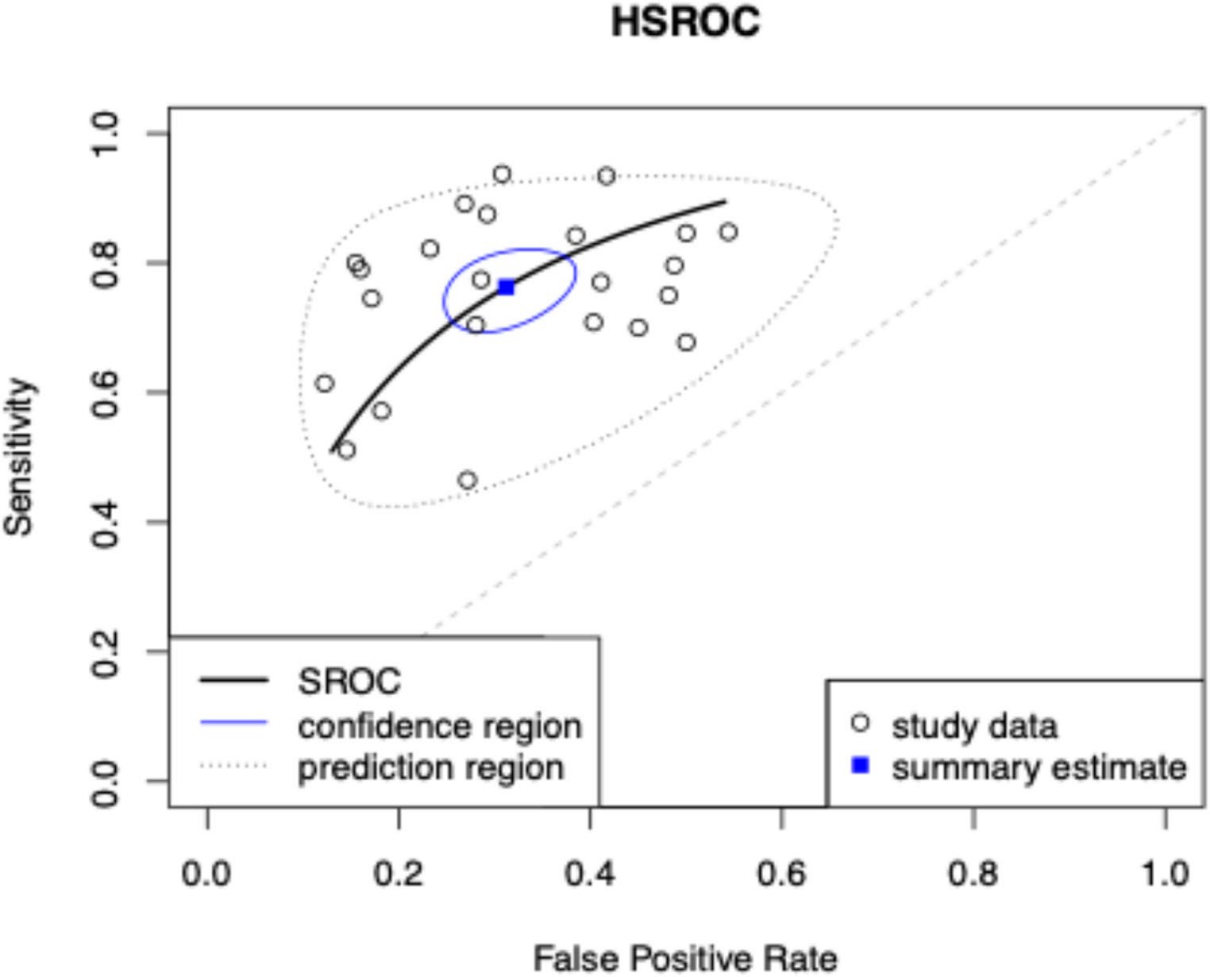
Hierarchical summary receiver operating characteristic curve (hsROC) from the bivariate model for diagnostic accuracy of TCCL to predict extraprostatic extension

Pooled sensitivity and specificity for the 23 studies were 76.3% (95% CI 70.8– 81.0%) and 68.8% (95% CI 63.0– 74.1%) respectively with an AUC of 0.787 for the hierarchical summary ROC curve. (Fig. 4) Deeks’ funnel plot asymmetry test showed no significant publication bias (*P* = 0.79).

## Meta-regression

Meta-regression analysis was performed to explore heterogeneity, using the following parameters: distribution of ISUP grades on prostatectomy specimen, reader experience, TCCL measurement method, and method used to set TCCL threshold. (Table 4A) The threshold method was a significant factor affecting the heterogeneity in sensitivity (*P* = 0.008). Studies set TCCL threshold by using ROC analysis to maximize Youden’s J index had a greater sensitivity (79.2% vs. 66.1%). Other parameters assessed did not significantly affect heterogeneity.

Pairwise comparisons of diagnostic performance at different TCCL thresholds did not show a statistically significant difference (Table 5) The pooled sensitivity estimate remained moderately high (73–76%) at all thresholds, however, the specificity increased from 68.5 to 73.4% with increasing thresholds (till 14 mm).

At thresholds of 10 mm and 14 mm, data from ≥ 5 studies were available and separate bivariate models were fit to compare diagnostic accuracy at these two thresholds. (Table 6) The differences in summary estimates were not statistically significant. Sensitivity was similar at both thresholds (72.5% and 73.3%), but the specificity at 14 mm (74.1%) was greater than at 10 mm (69.9%). The area under sROC curve, diagnostic odds ratio and likelihood ratios were more favorable at 14 mm threshold.

**Table 4:** Meta-regression analysis for diagnostic accuracy stratified by study characteristics and TCCL cutoff. Meta-regression including study parameters

Parameter		*n*	Sensitivity, % [95% CI]	*P* value	Specificity, % [95% CI]	*P* value
Proportion of ISUP ≥ 4	< 20%	11	79.3 [71.55–85.41]	0.113	68.3 [60.18–75.49]	0.789
	≥ 20%	9	70.5 [62.47–77.38]		70.1 [59.82–78.69]	
Proportion of ISUP ≤ 1	< 20%	12	74.6 [67.02–80.91]	0.231	67.4 [58.24–75.41]	0.367
	≥ 20%	6	80.2 [75.78–83.93]		73.7 [63.34–81.96]	
Reader Experience	< 10 y	7	77.5 [70.33–83.29]	0.695	66 [54.38–76.04]	0.822
	≥ 10 y	11	80 [74.14–84.78]		64.2 [56.45–71.26]	
Measurement method	Curvilinear	17	77 [69.98–82.82]	0.784	69.7 [63.13–75.53]	0.809
	Linear	3	79.7 [65.18–89.14]		67.4 [56.18–76.89]	
Threshold method	Youden index	17	79.2 [74.5–83.23]	0.008	69 [61.79–75.43]	0.945
	Other	6	66.1 [52.5–77.45]		68.5 [58.05–77.33]	

**Table 5:** Meta-regression including pairwise comparisons at multiple TCCL cut-offs

Cutoff	*n*	Sensitivity, % [95% CI]	*P* value	Specificity, % [95% CI]	*P* value
≤ 10 mm	8	76.2 [63.3–85.7]	0.855	69.5 [61.4–76.6]	0.865
> 10 mm	15	75.9 [70.7–80.4]		68.5 [60.5–75.6]	
≤ 11 mm	10	79.6 [68.8–87.3]	0.346	66.3 [58.– 73.8]	0.442
> 11 mm	13	72.8 [68.5–76.7]		70.9 [62.7–77.9]	
≤ 12 mm	11	78.6 [68.5–86.1]	0.493	65 [56.8–72.3]	0.203
> 12 mm	12	72.9 [68.3–77.1]		72.2 [64.1–79.1]	
< 13 mm	12	78.4 [69.3–85.3]	0.489	64.4 [57.– 71.2]	0.1
> 13 mm	11	72.5 [67.5–77]		73.4 [65–80.4]	
≤ 14 mm	17	76.9 [70.2–82.5]	0.744	67.1 [59.6–73.7]	0.287
> 14 mm	6	74 [64.1–81.9]		73.4 [65.9–79.7]	

**Table 6:** Pooled estimates for studies with 10 mm (6) and 14 mm (5) TCCL cutoff

Cutoff	*n*	Sensitivity, % [95% CI]	Specificity, % [95% CI]	AUC	DOR	posLR	negLR
10 mm	6	72.5 [56.3–84.4]	69.9 [58.9–79]	0.764	6.5 [3.1–12.1]	2.42 [1.76–3.31]	0.40 [0.24–0.61]
14 mm	5	73.3 [64.9–80.2]	74.1 [55.4–86.9]	0.775	8.31 [4.08–15.10]	2.99 [1.74–5.20]	0.37 [0.29–0.46]

## Discussion

This meta-analysis shows that TCCL on MRI has reasonable pooled sensitivity and specificity of 76% and 69% respectively for predicting ECE at histopathology in prostate cancer patients. We also found that an optimal cut-off for of TCCL for predicting ECE is difficult to determine, similar to prior meta-analysis by Kim et al. [36]. While sensitivity is similar for various cut-offs analyzed in this study, specificity increased with larger cut-offs. While sensitivity remained moderately high for all thresholds, specificity at 14 mm threshold was greater (threshold close to 15 mm suggested by PI-RADS v1.0) than that at 10 mm (cutoff suggested by PIRADS v2.1). Further, 14 mm threshold yielded a greater diagnostic odds ratio and better likelihood ratios, compared to 10 mm threshold.

The accuracy of mpMRI for predicting ECE varies widely and the inconsistency in accuracy is attributed to subjective assessment of secondary signs on MRI [8]. Assessment of ECE therefore varies with experience of the radiologist and often associated with poor inter-observer agreement despite addition of DWI and DCE imaging. Further, there are concerns about real-world accuracy of mpMRI for staging of localized prostate cancer [41]. A multi-institutional study demonstrated poor positive and negative predictive values in predicting ECE [42]. TCCL is an objective criterion which has the potential to decrease heterogeneity in reporting of ECE with better accuracy [15, 21, 43]. Ahn et al. reported that TCCL yielded greatest AUC for EPE compared capsular morphology, tumor dimension or arc-dimension ratio (TCCL divided by tumor dimension) [43]. In this study, a 10 mm threshold demonstrated sensitivity of 77% and specificity of 68%. TCCL has been shown to be an independent predictor of pathological extra-prostatic extension, pathological positive lymph nodes and biochemical recurrence [16]. Published studies have shown good inter-reader agreement for TCCL [13, 22].

Accordingly, the 14 mm threshold reported in this meta-analysis can be used to develop objective likelihood scores for predicting ECE on prostate mpMRI in contrast to previously reported subjective assessment of secondary signs. Furthermore, since clinical factors like positive digital rectal examination and pathologic features like cribriform histology and perineural invasion on biopsy specimens also predict ECE on final radical prostatectomy specimens, there is potential to include these factors into the scoring system to refine the prediction of ECE on mpMRI.

Though TCCL is a useful objective parameter to predict ECE, there is no uniform consensus on the threshold TCCL which can predict ECE. The current recommendation of tumor-capsule abutment of > 1 cm as a staging criterion in PI-RADS v2.1 is not sufficiently supported by existing literature. While the pooled sensitivity and specificity in this meta-analysis based on 23 eligible studies was 76% and 69%, respectively, the sensitivity and specificity in the individual studies varied widely according to the TCCL cut off used.

The lack of an optimal cut off for TCCL to predict ECE has been shown in a study. Bakir et al. tried to determine optimal cutoffs for TCCL at pathology and MRI to predict EPE and correlate MRI- and pathology-based TCCL [31]. The study did not find an optimal threshold when all ISUP grades obtained from RP specimens were analyzed together or individually. However, the authors noted that pathology based TCCL threshold associated with EPE decreased as the ISUP grade group increased. Similarly, the study also did not find relation between MRI-based TCCL thresholds and EPE for all biopsy-derived ISUP grade groups except for a threshold of 16 mm in low ISUP grade groups. Independent of the ISUP grades, the study found an MRI-based TCL threshold of 15–16 mm to be useful with a sensitivity of 71% and specificity of 60%. Interestingly, the study found strong correlation between MRI- and pathology-based TCCL, with stronger correlation in higher ISUP grades.

The wide range of TCCL thresholds can be partly explained by the variations in study cohorts, technical and interpretation variations. More importantly, the explanation for this probably lies in the fact that ECE is a continuum and not a simple binary observation [21]. Prostate cancer is inherently heterogeneous and the focal Gleason score at the tumor capsule junction may determine the presence or absence of ECE. The difference in grade groups of the published studies may explain the difference in thresholds. TCCL threshold can also vary with location of primary tumor, which was assessed in two studies [18, 32]. Park et al. showed no difference in the association of TCCL with ECE according to tumor location, whereas Matsumoto et al. showed posterior location was associated with higher probability of EPE at the same TCCL value. However, this was likely because of lower average Gleason scores in the anterior tumors in that study. Using a low threshold of TCCL to raise concern for potential ECE like 10 mm as recommended by PI-RADS v2.1 and some of the studies included in this metanalysis seems prudent to decrease false negative rates and improve true positive rates even at the risk of low specificity [12, 13, 18, 19]. However, given the improved surgical and radiation oncology techniques, higher specificity offered by a TCCL cutoff of 14 mm can minimize false positive rate and ensure unnecessary treatment strategies are not initiated.

In addition to TCCL, other quantitative methods show promise in improving prediction of ECE. A PI-RADS score of 5 on mpMRI maybe associated with ECE. However, the included studies did not include separate analysis independent of TCCL. Quantitative histology is another well studied objective criterion that can predict ECE [44]. Further, there is a potential role of 68Ga-PSMA PET/CT in diagnosis and staging of prostate cancer using standardized uptake values (SUVmax) [45, 46].

There are several limitations to this study. First, there was significant heterogeneity in the included studies in terms of study and patient characteristics. Though all studies had RP specimens as reference standard, the Gleason scores and Grade groups were not uniformly reported. Previous studies suggest that the optimal threshold varies with the ISUP grade. In this meta-analysis we could not assess this association due to lack of raw data. The scanner strength and number of readers in individual studies were also not uniform. In addition, most of the included studies (20 of 23) were retrospective, which may introduce selection bias. Despite these limitations, we believe that the results of this meta-analysis will help in clarifying to certain extent the conundrum of TCCL in predicting ECE.

In conclusion, TCCL on MRI is a useful objective criterion to predict ECE with reasonable pooled sensitivity and specificity. Though we did not find an optimal threshold which can predict ECE, using the cutoff of 14 mm was associated with higher specificity and yielded a greater diagnostic odds ratio and better likelihood ratios, compared to 10 mm threshold. Future studies can use this threshold to develop objective likelihood scores for predicting ECE on prostate mpMRI especially when combined with preoperative factors like positive DRE and pathologic features like cribriform histology and perineural spread on biopsy specimens.

## Data Availability

No datasets were generated or analysed during the current study.
